# *Durvillaea antarctica*: A Seaweed for Enhancing Immune and Cardiometabolic Health and Gut Microbiota Composition Modulation

**DOI:** 10.3390/ijms241310779

**Published:** 2023-06-28

**Authors:** Marion Guerrero-Wyss, Caroline Yans, Arturo Boscán-González, Pablo Duran, Solange Parra-Soto, Lissé Angarita

**Affiliations:** 1 Escuela de Nutrición y Dietética, Facultad para el Cuidado de la Salud, Universidad San Sebastián, Valdivia 5090000, Chile; 2 Escuela de Nutrición y Dietética, Facultad de Salud, Universidad Santo Tomás, Puerto Montt 5480000, Chile; cyans@santotomas.cl; 3 Facultad de Medicina, Escuela de Medicina, Universidad del Zulia, Maracaibo 4001, Venezuela; arturojboscanmd@gmail.com; 4 Centro de Investigaciones Endocrino-Metabólicas, Escuela de Medicina, Universidad del Zulia, Maracaibo 4001, Venezuela; pabloduran1998@gmail.com; 5 Departamento de Nutrición y Salud Pública, Facultad Ciencias de la Salud y de los Alimentos, Universidad del Bío-Bío, Chillán 3780000, Chile; sparra@ubiobio.cl; 6 Escuela de Nutrición y Dietética, Facultad de Medicina, Universidad Andres Bello, Concepción 4260000, Chile; lisse.angarita@unab.cl

**Keywords:** *Durvillaea antarctica*, microbiota, metabolic syndrome, cardiovascular risk, seaweed

## Abstract

*Durvillaea antarctica* is the seaweed that is the most consumed by the Chilean population. It is recognized worldwide for its high nutritional value in protein, vitamins, minerals, and dietary fiber. This is a narrative review in which an extensive search of the literature was performed to establish the immunomodulator, cardiometabolic, and gut microbiota composition modulation effect of *Durvillaea antarctica*. Several studies have shown the potential of *Durvillaea antarctica* to function as prebiotics and to positively modulate the gut microbiota, which is related to anti-obesity, anti-inflammatory, anticancer, lipid-lowering, and hypoglycemic effects. The quantity of *Bacteroides* was negatively correlated with that of inflammatory monocytes and positively correlated with the levels of several gut metabolites. Seaweed-derived polysaccharides modulate the quantity and diversity of beneficial intestinal microbiota, decreasing phenol and p-cresol, which are related to intestinal diseases and the loss of intestinal function. Additionally, a beneficial metabolic effect related to this seaweed was observed, mainly promoting the decrease in the glycemic levels, lower cholesterol levels and cardiovascular risk. Consuming *Durvillaea antarctica* has a positive impact on the immune system, and its bioactive compounds provide beneficial effects on glycemic control and other metabolic parameters.

## 1. Introduction

*Durvillaea antarctica*, also called “cochayuyo”, is the seaweed that is the most consumed by the Chilean population; its consumption represents approximately 0.5 kg per capita. It grows particularly on the Chilean and New Zealand coasts. Seaweeds are recognized worldwide for their high nutritional value in protein, vitamins, minerals, and dietary fiber [1]. Studies indicate that seaweed products will increase from USD 4.700 million to USD 6.400 million by 2026, due to its multiple health properties [1]. Cochayuyo also stands out for its low caloric content and its high content of omega-3 essential fatty acids, as well as for being a source of numerous bioactive compounds with beneficial activity for the body. There are reports that over 50% of the dry weight of this food corresponds to dietary fiber [2]. These characteristics represent an ideal opportunity to incorporate this food and its bioactive derivatives into diet therapy in metabolic syndrome (MS).

A regular diet with a high total dietary fiber content, which is the most important component of *Durvillaea antarctica*, contributes to reducing LDL cholesterol and plasmatic triglycerides [3]. Several studies indicated that soluble fiber can reduce serum cholesterol and glucose concentrations, as well as lower blood pressure, promote weight loss, improve insulin sensitivity and, consequently, contribute to improving metabolic syndrome and reducing morbidity and mortality associated with cardiovascular risk [4].

Metabolic syndrome is a group of disorders that occur in parallel and increase cardiovascular risk, that is, it involves a greater probability of developing coronary disease, stroke, and type 2 diabetes mellitus (T2DM) (10.4% national prevalence) [5]. The national prevalence of this syndrome reaches approximately 40.1% [5]. These disorders involve arterial hypertension (27.6%) and elevated cholesterol levels (27.8%) and plasma triglycerides (38.5%), but the basis of all of them is undoubtedly excess weight (73.2%) that generates insulin resistance and an increase in visceral adiposity, with its consequent increase in the cardiovascular risk [5,6].

The human intestine is inhabited by billions of microorganisms that constitute a dynamic ecosystem that can be related to health and disease processes. The gut microbiota is modified, among other factors, by variations and types of habitual diet, which generate potential dietary therapeutic strategies to manipulate the microbiome diversity, composition, and stability [7].

This review article aims to provide a comprehensive overview of the current and novel findings regarding the therapeutic potential of brown algae, specifically *Durvillaea antarctica*, in immune, intestinal, and cardiometabolic diseases. By conducting a comprehensive review and analysis of the literature, including the consultation of sources and the selection and discussion of evidence, from September 2022 to May 2023, we elucidate the mechanisms through which the components of this algae exhibit immunomodulatory effects in specific pathological conditions. Additionally, we explore its impact on the modulation of the intestinal microbiota, which has emerged as a crucial factor in the management of chronic and systemic diseases. Furthermore, we examine how *Durvillaea antarctica* can influence cardiometabolic risk factors, given its recognized antioxidant, anti-inflammatory, and regulatory properties in lipid and carbohydrate metabolism.

In assessing the critical aspects mentioned above, it is crucial to emphasize the potential for obtaining significant results from this research. The investigation into the immunomodulatory potential of *Durvillaea antarctica* presents promising prospects for its application as a therapeutic agent in immune-related disorders. Moreover, the emerging understanding of its impact on the intestinal microbiota offers a fresh perspective on managing chronic and systemic diseases, highlighting the significance of a comprehensive approach.

Furthermore, the recognition of *Durvillaea antarctica*’s role in regulating cardiometabolic risk factors holds great promise for interventions targeting metabolic syndrome and related conditions. These findings open up exciting possibilities for achieving meaningful outcomes and advancing our understanding of the potential benefits of *Durvillaea antarctica* in various health contexts.

### 1.1. Immunomodulatory Effect of Durvillaea antarctica

**Figure 1:**
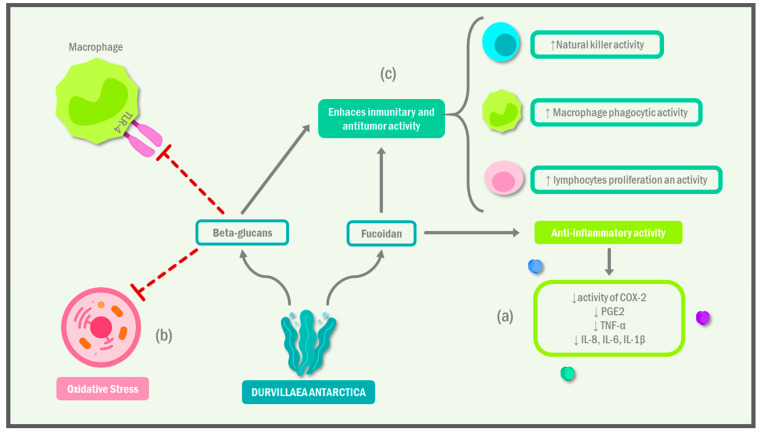
Immunomodulatory effect of *Durvillaea antarctica*. The components of *Durvillaea antarctica* exhibit the following immunomodulatory mechanisms: (**a**) they can act as anti-inflammatory as they modulate immune cells to decrease the synthesis and release of proinflammatory cytokines; (**b**) they inhibit the production of free radicals, and thus, decrease oxidative stress on cells; and (**c**) they possess antitumor activity and can contribute to the activation of natural killer cells, lymphocytes, and macrophages under situations that merit immune reactivity. IL: interleukin; COX: cyclooxygenase; TNF-α: tumor necrosis factor alfa; PGE: prostaglandins.

Algae were shown to contain various bioactive compounds, such as polysaccharides, polyphenols, and proteins, that have potential health benefits. These compounds were found to possess immunomodulatory properties, meaning that they can stimulate or suppress immune responses as needed [8]. One of the most studied immunomodulatory compounds found in algae is fucoidan, a sulfated polysaccharide that is found in brown algae [9,10]. Fucoidan was shown to modulate immune function by regulating the production of cytokines and chemokines, which are signaling molecules that help to regulate immune responses. In a recent study, Lee et al. [11] found that treatment with *Laminaria japonica* extract attenuated the activity of COX-2 and decreased the production of PGE2, TNF-α, and IL-8 in the same in vitro model. Similarly, another study showed that treatment with fucoidan decreased the levels of IL-1β and IL-6 and suppressed the synthesis of inflammatory chemokines [12]. Studies found that fucoidan can enhance the activity of immune cells, such as natural killer cells and macrophages, which play an important role in fighting infections and cancer (Table 1). Moreover, fucoidan was found to possess anti-inflammatory properties that contribute to its immunomodulatory effects (Figure 1) [13].

A group of researchers in 2013 determined the immunomodulatory effect of β-1,3/1,6-glucan in marine algae from the southern hemisphere. Mouse cells were exposed to *Durvillaea antarctica* extract (50, 100, 250, and 500 µg/mL). The results highlight that β-glucan present in *D. antarctica* induced a 16.9% increase in the activation of CD19+B lymphocytes compared to the control. The optimal concentration for maximum immunomodulatory activity was 100 µg of DA extract/mL [14]. Similarly, in a study on a macrophage cell line, an increase in macrophages with proinflammatory activity infiltrating the tumor was observed, prioritizing the systemic and intratumoral cell composition and, consequently, the antitumor effect in multiple types of tumor models. The authors state that highly purified, water-soluble, and low molecular weight β-1,3/1,6-glucan (BG136) present in *Durvillaea antarctica* is a potent immune stimulator and a valuable polysaccharide in cancer immunotherapy [15].

Another study published in 2018 indicates that a low molecular weight β-glucan present in *Durvillaea antarctica* has an immunomodulatory effect on macrophages (RAW264.7) through the Toll-like receptor 4 (TLR4) [16]. On the other hand, extracts of *Durvillaea antarctica* have shown significant anti-herpetic activity against HSV-1 and HSV-2 viruses. The evaluation of the extracts of this alga as a topical formulation in an animal model infected with HSV-1 revealed that the extracts reduced the severity and duration of the lesions to a greater extent than acyclovir [17].

Likewise, an investigation was carried out in four types of marine algae, among which *Durvillaea antarctica* was analyzed. The study consisted of the extraction, structural characterization, and the potential antioxidant activity of the polysaccharides present in each one. The results show that the polysaccharides exhibited concentration-dependent antioxidant activity. The polysaccharides present in *Durvillaea antarctica* also had an inhibitory effect on the free radicals [18]. In addition, in a study, it was shown that *Durvillaea antarctica* polysaccharides increased cell viability and inhibited EV71 virus infection. Additionally, these compounds reduced the population of apoptotic cells through the inhibition of the P53 and STAT1 signaling pathways. This study determined that polysaccharides present in cochayuyo could effectively prevent Vero cells from EV71 viral infection [19]. All of these results promote the hypothesis of a potential immunomodulatory effect of this seaweed not only in preclinical models, but also in humans.

The in vitro studies conducted by Qin et al. [20] revealed that DAP4 (*Durvillaea antarctica* polysaccharide subfraction 4), a fucoidan extracted from *Durvillaea antarctica*, exhibits exceptional immunomodulatory activities. DAP4 promotes the proliferation of RAW264.7 cells as well as spleen lymphocytes, and also enhances the phagocytic activity of macrophages. Additionally, DAP4 increases the production of nitric oxide and the vitality of natural killer cells. The findings indicate that DAP4 has significant immune-enhancing potential and could be a promising source of immunomodulatory fucoidan with a unique structure. These results highlight the potential of DAP4 as a candidate for the development of new therapeutic agents for immune-related diseases.

**Table 1:** Immunomodulatory Effects of *Durvillaea antarctica*: A Comprehensive Summary for Research.

Author (REF)	Phytochemical Compound Tested	Rationale	Results–Key Findings
Lee et al. [11]	*Laminaria japonica* extract (LJE)	Study the mitigating LJE on inflammatory reactions of the skin when applied to UVB-induced nc886-PKR pathways, and the regulatory effect of nc886 on the PKR signal transduction channel induced by UVB.	LJE inhibited UVB-induced inflammation in human keratinocytes. LJE treatment reduced the expression of MMP-9, PGE2, IL-8, and TNF-α, and it also inhibited the phosphorylation of p38, SAPK/JNK, c-Jun, ATF-2, and PKR under basal and UVB conditions.
Castillo et al. [17]	*Macrocystis pyrifera* and *Durvillaea antarctica* aqueous extracts	Evaluate the potential antiviral properties of extracts obtained from two brown macroalgae against both HSV-1 and HSV-2 in humans (HeLa cells) and primary human gingival fibroblasts.	Algae extracts inhibited the growth of both viruses in a dose-dependent manner. The algae extracts also reduced the binding of HSV-1 and HSV-2 to HeLa cells, and decreased the expression of the viral proteins gB and gD.
Xu et al. [19]	*D. antarctica* polysaccharide (DAPP)	Validate how DAPP inhibits EV71 to induce the apoptosis of Vero cell.	DAPP had no toxicity on Vero cells at the concentration of 250 μg/mL. DAPP inhibited the proliferation of EV71 virus in a dose-dependent manner, inhibited the Vero cells’ apoptosis induced by EV71 via P53 signaling pathway, and decreased the expression of proinflammatory cytokines.
Qin et al. [20]	Sulfated polysaccharide 4 of *D. antarctica* (DAP4)	Isolation and purification of DAP4 via methylation analysis and NMR spectrometry analysis. Evaluation of immunomodulatory activity in vitro, including lymphocyte proliferation, phagocytic activity of macrophages, NO production, and NK cell cytotoxicity in vitro.	DAP4 had immunomodulatory activity in vitro. DAP4 was non-toxic to RAW264.7 cells at concentrations of up to 400 μg/mL. DAP4 also enhanced the phagocytic activity of RAW264.7 cells. DAP4 increased the production of NO by RAW264.7 cells. DAP4 also enhanced the proliferation of splenocytes in response to ConA and LPS. DAP4 increased the cytotoxicity of NK cells against YAC-1 cells.

Abbreviations: Noncoding RNA nc886 (nc8886); matrix metalloproteinase-9 (MMP-9); cyclooxygenase-2 (COX-2); prostaglandin E2 (PGE2); interleukin-8 (IL-8); tumor necrosis factor α (TNF-α); herpes simplex virus (HSV-1/2); stress-activated protein kinases (SAPK)/Jun amino-terminal kinases (JNK); transcription factor Jun (c-JUN); activating transcription factor 2 (ATF2); protein kinase R (PKR); enterovirus 71 (EV71); nitric oxide (NO); Concanavalina A (ConA); lipopolysaccharide (LPS).

### 1.2. Durvillaea antarctica as a Cornerstone for Gut Microbiota Modulation

The cochayuyo is a seaweed that, in its composition, stands out for its contribution of β-glucans; polysaccharides such as fucoidan, laminarin, alginate, ulvan, and porphyran are unique to seaweeds. It was reported that these dietary components have biological activity associated with anticancer, antidiabetic, and anti-inflammatory functions, and have an immunomodulatory effect [21]. Evidence suggests that β-glucans could have a significant impact on changes in the microbiota and have an improvement on human health. In this vein, several studies have shown their potential prebiotic activity and their ability to positively modulate the gut microbiota. Prebiotics enhance bacterial populations, and their production of short-chain fatty acids (SCFAs), which are the energy source for gastrointestinal epithelial cells, often provide protection against pathogens, influence immunomodulation, and induce apoptosis of colon cancer cells [22].

A study developed by He et al. [23] examined the impact of high doses of a combination of deep-sea water and fucoidan (H-CDF) on the gut microbiota of rats with T2DM using 16S rDNA sequencing. The results demonstrate that H-CDF significantly increased bacterial diversity and restored the abundance and diversity of gut microbiota to normal levels. At the phylum level, Firmicutes and Bacteroidetes were the dominant microflora in all groups. However, after H-CDF intervention, the abundance of Firmicutes increased to normal levels, and the F/B ratio increased significantly. The study suggests that H-CDF could be a potential therapeutic intervention for T2DM, but further research is needed to determine the mechanisms by which H-CDF regulates gut microbiota and its long-term effects.

**Figure 2:**
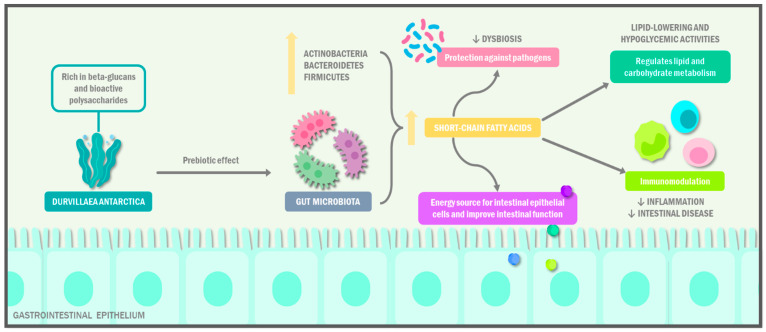
Prebiotic effect of *Durvillaea antarctica*.

In recent research, the quantity of *Bacteroides* was negatively correlated with that of inflammatory monocytes and positively correlated with the levels of several gut metabolites after the sodium alginate (SA) supplementation, a seaweed-derived dietary fiber [24]. The consumption of seaweed is associated with an increase in the concentrations of SCFAs, producing a modulation of epithelial cells and leukocytes in the immune system. Seaweed-derived polysaccharides modulate the quantity and diversity of beneficial intestinal microbiota, generating a decrease in phenol and p-cresol, which are related to intestinal diseases and the loss of intestinal function. Bai et al. [25] studied the in vitro effects of alginate on the microbiota, observing that it favors the proliferation of beneficial *bifidobacteria* and decreases pathogenic bacterial strains, increasing the colonic fermentation time and the consequent production of SCFAs (Figure 2).

*D. Antarctica* possesses numerous phytochemicals with prebiotic properties, including beta-glucans and polysaccharides; this effect positively modulates the gut microbiota, promoting an increase in beneficial bacteria, which, in turn, contributes to the protection against intestinal pathogens, promotes intestinal cell turnover and physiology, regulates carbohydrate and lipid metabolism, and reduces local and systemic inflammation through immunomodulatory mechanisms.

The sulphated polysaccharides present in seaweeds have anti-obesity, anti-inflammatory, anticancer, lipid-lowering, and hypoglycemic activities, due to their activity on the intestinal microbiota, which promotes the relationship between Bacteroidetes and Firmicutes, inhibits proinflammatory bacteria, and regulates lipid and carbohydrate metabolism through the production of SCFA [26]. On the other hand, Bermano et al. [27] analyzed the prebiotic potential of seaweed in rats, obtaining a lower body weight and serum triglyceride concentration compared to the control group; in addition, in humans, it is evident that the consumption of seaweed increases the frequency of defecation, favoring the concentration of bifidobacteria.

A study conducted by Yang et al. [28] on hamsters determined that oligosaccharides from seaweed have hypoglycemic and lipid-lowering effects by stimulating the insulin secretion and improving glucose tolerance. In addition, they demonstrated that hamsters that were fed a diet that is rich in fat and sucrose, by consuming oligosaccharides from the algae, had an increased population of Bacteroidetes and decreased plasma glycaemia. Therefore, the increase in Firmicutes, together with the decrease in Bacteroidetes, are significantly related to plasma glucose concentrations. Similarly, Siddiqui et al. [29] showed that administering a crude seaweed polysaccharide to rats with type 1 diabetes mellitus (T1DM) improved diabetes symptoms, decreased body weight, and improved fasting blood glucose and pancreatic β cells. Although the mechanism is not fully established, the rats presented an increase in the population of beneficial bacteria of the intestinal microbiota, such as Lactobacillus and Bacteroidetes (Table 2).

Another beneficial component found in seaweed is SA, which has antitumor, anticoagulant, and immunomodulatory effects. When evaluating the effect of SA on immunosuppressed rats, the restoration of impaired immune functions, the decrease in T lymphocytes, and the increase in the secretion of serum immunoglobulins and proinflammatory cytokines are obtained, promoting the increase in beneficial bacteria such as Lactobacillus, which helps with inflammation and immunity [30]. At the same time, another study shows that when using SA in rats with obesity and metabolic syndrome that are fed a high-fat diet, it reduces weight gain, the accumulation of fat in the liver, and inflammation, and improves the intestinal microbiota, through the increase in Bacteroidetes and AGCC [31].

On the other hand, Wang et al. [32] studied the side effects of taking antibiotics, which alter the intestinal barrier, causing a decrease in both the immune function and drug efficacy. After administering antibiotics to mice, the authors observed that by combining them with fucoidan, a polysaccharide that is present in marine algae, the symptoms of inflammatory bowel disease were alleviated by avoiding alterations in the colonic tissue. In addition, the microbiota dysbiosis decreased by increasing the number of beneficial bacteria and promoting the synthesis of IL-10, which is an anti-inflammatory cytokine. In the same vein, Deng et al. [33] observed that other benefits of fucoidan polysaccharide are the reduction in blood glucose, improved insulin sensitivity, reduced hepatic oxidative stress, improved hepatocyte steatosis, and increased beneficial bacteria of the intestinal microbiota such as *Verrucomicrobia* and *Akkiermansia muciniphila*.

Additionally, the consumption of seaweeds generates an increase in immunoglobulin levels, both immunoglobulin A and G, in rats with supplemented diets. Alternatively, they stimulate the growth of beneficial bacteria in the colon and a slight decrease in pathogenic bacteria. Regarding colonic metabolites, a significant increase in SCFAs was observed, specifically acetic, propionic, and butyric acids, and a change in colonic morphology in relation to epithelial cells and intestinal mucosa [34]. Maintaining the balance of epithelial cells is crucial for the function of the intestinal mucosal barrier. Marine algae-derived bioactive peptides, such as phycobiliproteins, which are pigmented proteins involved in capturing light energy for photosynthesis, along with glycoproteins containing “cellulose binding domains”, suggest a potential role in the cell wall structure and adhesion. Additionally, phycolectins, which are lectins that recognize and bind to specific carbohydrates, and mycosporine-like amino acids, which act as natural sunscreens against UV radiation, are also present. These diverse bioactive peptides exert their effects by stimulating the epidermal growth factor (EGF), leading to the enhanced growth, proliferation, and differentiation of intestinal epithelial cells. By modulating these cellular processes, these bioactive peptides contribute to the overall health and well-being of the host [35]. Reilly et al. [36] investigated the effects of marine algae on the intestinal morpho-physiology of pigs, discovering that the consumption of an algae extract provided a lower population of enterobacteria, bifidobacteria, and enterobacteria in the cecum and colon; increased the proportion of butyric acid; decreased the concentration of ammonia; and increased the expression of interleukin (IL)-8 mRNA.

Compositional changes in the overall gut microbiota are significantly dependent on several markers related to metabolic syndrome, including body weight, glucose–insulin homeostasis, endotoxemia-induced inflammation, and intestinal barrier integrity. According to Cheng et al. [37], although the evidence of gut microbiota is insufficient, the relationship between the factors involved in its maintenance and mediation in healthy subjects proves to be relevant in human health and disease. Significant metabolic pathologies, such as diabetes, are known to be partially caused by the imbalance of interactions between the host and the gut microbiota. The gut microbiota in individuals with T1DM is characterized by reduced bacterial and functional diversity, as well as low bacterial community stability [38]. A study presented by Du Preez et al. [39] showed that 5% *S. Siliquosum* supplementation in male Wistar rats with diet-induced metabolic syndrome decreased body weight and retroperitoneal fat due to an increase in the gut microbiota, which likely complements the prebiotic actions of alginates that are present in some brown algae. Other *Sargassum* species show identical responses related to gut microbiota regulation.

**Table 2:** *Durvillaea antarctica* as a key player in gut microbiota modulation.

Author (REF)	Phytochemical Compound Tested	Rationale	Results–Key Findings
He et al. [23]	Combination of deep-sea water (DSW) and/or fucoidan (CDF)	The combined effect of DSW and fucoidan was investigated on a T2DM rat model induced by a high-fat diet and streptozocin injection. Fecal metabolomics and 16S rDNA analysis were used to explore the relationship between these interventions and identify potential metabolic pathways.	CDF was more effective than DSW or fucoidan alone in improving blood glucose, lipid levels, and histopathological changes in T2DM rats. CDF also enhanced the phosphorylation of Akt and GSK3β, which are important steps in insulin signaling. Fecal metabolomics and 16S rDNA analysis showed that CDF altered the composition of gut microbiota and metabolic pathways.
Bai et al. [25]	Alginate	Alginate overproducing mutant of *P. aeruginosa* was obtained through transposon mutagenesis libraries. The in vitro functions of human gut microbiota in degrading seaweed and mutant *Pseudomonas* alginates were comparatively studied.	Both bacterial and seaweed alginates were found to be completely degraded by fecal bacteria isolated from study volunteers. Moreover, their regulatory function on gut microbiota was similar, as they promoted the proliferation of beneficial bifidobacteria while reducing the abundance of pathogenic bacterial strains.
Siddiqui et al. [29]	Crude polysaccharide from seaweed, *Dictyopteris divaricata* (CDDP)	The impact of streptozotocin-induced T1DM on gut barrier permeability and gut microbiota dysbiosis.	CDDP treatment increased beneficial bacteria (Firmicutes, Bacteroidetes, Lactobacillus) via 16S rRNA sequencing. Immunohistological analysis confirmed CDDP’s anti-inflammatory effects, restoring colon morphology and maintaining gut structure and barrier permeability.
du Preez et al. [39]	*Sargassum siliquosum* extract	Evaluated the impact *of S. siliquosum* on metabolic syndrome parameters, including heart/liver function, plasma biochemistry, glucose/insulin responses, body composition, and gut microbiota composition.	*S. siliquosum* decreased body weight, fat mass, abdominal fat deposition, liver fat vacuole size, and improved glucose tolerance and insulin sensitivity. *S. siliquosum* also increased the population of beneficial bacteria in the gut and reduced inflammation.

Abbreviations: Glycogen synthase kinase-3 beta (GSK-3 beta); serine/threonine-protein kinase (akt).

New evidence concerning the prebiotic capacities of seaweeds is related to the abundance of polysaccharide components, thanks to the insights on saccharolytic fermentation by the gastrointestinal microbiota. The results from original animal studies give encouraging data regarding the use of red seaweed galactans and brown seaweed glycans, such as alginates and laminarins [40]. Thus, Li et al. [41] showed that after an unsaturated alginate oligosaccharides (UAOS) treatment, the concentration and variety of the gut microbiota increased, represented by *Akkermancia* spp., *Lactobacillus* spp., *Bifidobacterium* spp., and *Saccharomyces* spp., with the potential of lowering body weight gain and improving glucose and lipid homeostasis. High-fat diet (HFD)-induced obesity markedly altered the percentage of the gut microbe phyla, while UAOS treatment significantly reversed this tendency, which suggests that UAOS can regulate the gut microbiota.

Likewise, Fu et al. [42] surveyed a new polysaccharide named ST-P2 from *Sargassum thunbergii*, a brown alga. ST-P2 fermentation significantly modulated the composition and growth of salutary colonic bacterial populations. The microbiome was examined at the phylum and genus levels; the dominant bacterial communities in the original fecal sample were Firmicutes, Bacteroidetes, Proteobacteria, and Actinobacteria. A significant increase in the salutary Bacteroidetes group combined with a significant decline in the dangerous Firmicutes group after ST-P2 supplementation were noted. Thus, ST-P2 is anticipated to be a functional component for health enhancement by modulating the gut health. In this way, it is demonstrated that certain DA phytochemicals, such as fucoidan, can possibly regulate the proliferation of beneficial bacteria such as those belonging to the phylum Bacteroidetes and, on the contrary, decrease the presence of potentially pathogenic bacteria of the phylum Firmicutes. Thus, the possible therapeutic potential of DA as a modulator of the gut microbiota is highlighted; however, it is pertinent to perform clinical studies to corroborate this effect in humans.

Another possible hypothesis that could be put forward is the relationship between DA and the activation of nucleotide oligomerization domain (Nod)-like receptors (NLRs). These are cytosolic receptors that are predominantly located in immune cells, and whose activation is associated with caspases signaling cascades, leading to the release of proinflammatory cytokines such as IL-1β and IL-18 [43,44]. Such cascade is generated once ligands related to intestinal dysbiosis states, such as microbe-associated molecular patterns (MAMPs), bind to NOD1/2 receptors [44,45]. Therefore, by combating intestinal dysbiosis thanks to its prebiotic effects, DA supplementation could reduce the activity of such receptors and, thus, decrease local and systemic inflammation.

Considering the above, the consumption of not only seaweed, but also other types of algae, have a significant impact on the structural and functional composition of the intestinal microbiota. These changes are associated with health and disease processes, in which immunomodulatory and anti-inflammatory mechanisms are mainly involved, as well as an improvement in various cardiometabolic health criteria. Additionally, further investigation of the impact of seaweed on gut microbiota is needed to establish this supplementation as a possible therapeutic tool for several diseases.

### 1.3. Cardioprotective Role of Durvillaea antarctica

**Figure 3:**
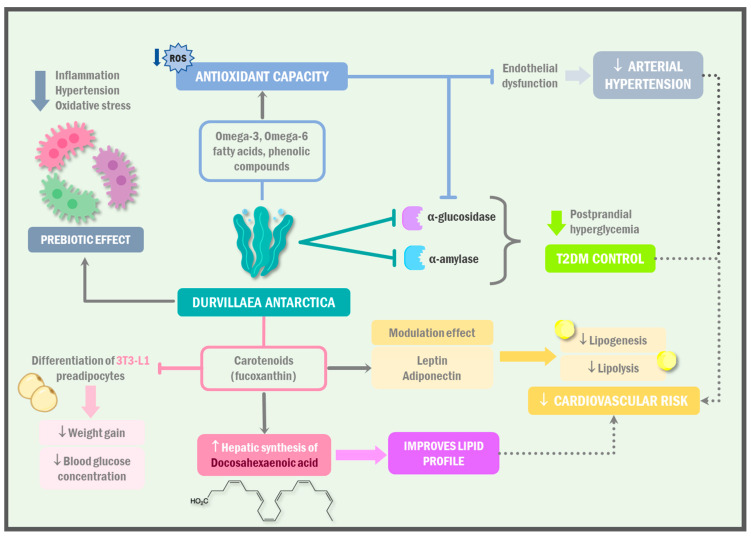
Cardiometabolic effect of *Durvillaea antarctica*.

Algae, particularly *Durvillaea antarctica*, are a vegetable food of marine origin with a high contribution of omega-3 and omega-6 fatty acids [46,47]. Even more important, cochayuyo stands out for its content of bioactive compounds, such as vitamins (C and E), carotenoids, and many phenolic compounds, which have shown antioxidant capacity [48,49,50] (Figure 3).

The illustration showcases the diverse health benefits of *Durvillaea antarctica*. This marine alga, which is rich in omega-3 and omega-6 fatty acids, contains bioactive compounds such as carotenoids, and phenolic compounds with potent antioxidant capacity. Notably, fucoxanthin plays a significant role in lipid metabolism, reducing cardiovascular risk by modulating leptin and adiponectin. Algae consumption inhibits enzymes related to hyperglycemia, controls T2DM, counteracts arterial hypertension, promotes weight loss, enhances thermogenesis, and improves the lipid profile. These findings support the potential of *Durvillaea antarctica* as a valuable dietary component. ROS: reactive oxygen species; T2DM: type 2 diabetes mellitus.

Among the carotenoids present in *Durvillaea antarctica*, fucoxanthin (FX) stands out, which is involved in lipid metabolism and modulating the action of leptin and adiponectin, thus reducing lipogenesis and lipolysis and decreasing cardiovascular risk [51,52]. In addition, in a study carried out by Lomartire et al. [53] in 2021, it was shown that fucoxanthin can make up to 30% of the dry weight of the algae. In humans, consuming the microalgae *Phaeodactylum tricornutum* (PT) led to an increase in the uptake of fucoxanthin, which is metabolized into fucoxanthinol (FXOH) and amarouciaxanthin A (AxA). The plasma levels of FX and its metabolites were measured in 22 participants before and after a two-week intervention with PT. It was found that FX was well absorbed, and the metabolites of FX were detected at higher concentrations in plasma than FX [54] (Table 3).

Alternatively, in a cohort study that evaluated the consumption of seaweed on cardiovascular risk in Japanese people of both the male and female genders, it was observed that the intake of seaweed was inversely associated with the risk of stroke in male subjects only (CI 0.42–0.94, *p* = 0.01) [55]. Another cohort study, carried out in the Japanese population, evaluated the correlation between the intake of seaweed and mortality from cardiovascular diseases; the results show that men and women who consumed seaweed daily had lower cardiovascular mortality than those who did not consume seaweed (CI 0.55–0.95, *p* = 0.72) [56].

### 1.4. D. antarctica as a Promising Therapeutic Dietary Agent for the Management of Metabolic Syndrome

Metabolic syndrome is a cluster of conditions that increase the risk of developing cardiovascular disease, type 2 diabetes, and other health problems [57]. The most important criteria for diagnosing metabolic syndrome include abdominal obesity, high blood pressure, hyperglycemia, and abnormal cholesterol levels. These criteria are used to identify individuals who are at high risk of developing these conditions and who may benefit from early intervention to prevent or delay their onset [58]. Recent studies demonstrated that metabolic syndrome affects over 30% of the adult population globally, with a particularly high prevalence in low- and middle-income countries. The increasing incidence of metabolic syndrome represents a significant public health concern, as it is associated with an elevated risk of cardiovascular disease and other chronic conditions. Given the potential impact of metabolic syndrome on health outcomes, it is critical to promote awareness of this condition and develop effective prevention and management strategies [59].

Metabolic syndrome is a complex disorder that involves several molecular mechanisms, including insulin resistance, chronic inflammation, oxidative stress, and mitochondrial dysfunction. Insulin resistance, a hallmark of metabolic syndrome, is characterized by impaired insulin signaling and reduced glucose uptake by insulin-sensitive tissues, such as muscle and adipose tissue. This results in increased blood glucose levels and compensatory hyperinsulinemia, which can further exacerbate insulin resistance and contribute to the development of metabolic syndrome [60].

Chronic inflammation also plays a critical role in the pathogenesis of metabolic syndrome. Inflammatory cytokines, such as interleukin-6 (IL-6) and tumor necrosis factor-alpha (TNF-α), are increased in individuals with metabolic syndrome, and they contribute to the development of insulin resistance and other metabolic abnormalities [61]. In addition, oxidative stress, which results from an imbalance between reactive oxygen species (ROS) production and antioxidant defense mechanisms, can also contribute to the development of metabolic syndrome. Increased ROS production can damage cellular components, including proteins, lipids, and DNA, leading to cellular dysfunction and insulin resistance [62].

Mitochondrial dysfunction, characterized by impaired mitochondrial function and biogenesis, was also implicated in the pathogenesis of metabolic syndrome. Reduced mitochondrial function can lead to decreased ATP production, which can impair insulin signaling and glucose uptake, contributing to the development of insulin resistance. Furthermore, dysfunctional mitochondria can produce more ROS, exacerbating oxidative stress and inflammation [63].

Marine algae, especially *Durvillaea antarctica*, have several bioactive compounds with great antioxidant capacity, which were shown to inhibit the enzymes α-glucosidase and α-amylase, causing a decrease in postprandial hyperglycemia, delaying starch hydrolysis, and thus, controlling T2DM, as demonstrated in the study by Pacheco et al. [64] where they evaluated the antioxidant effect and the inhibition capacity of the enzymes mentioned in six Chilean algae, finding that *Durvillaea antarctica* had the highest amount of polyphenols, antioxidant activity, and enzyme inhibition (Table 3).

Arterial hypertension, which is present in metabolic syndrome, could be counteracted with the consumption of algae, specifically *Durvillaea antarctica*, because it is an important source of prebiotics, specifically alginate, which acts on intestinal bacteria producing long-chain fatty acids; these compounds have anti-inflammatory and antioxidant activity that could counteract endothelial dysfunction, which is a cornerstone in arterial hypertension [22]. On the other hand, the peptides present in the seaweed inhibit the angiotensin-converting enzyme, presenting a hypotensive effect [65].

**Figure 4:**
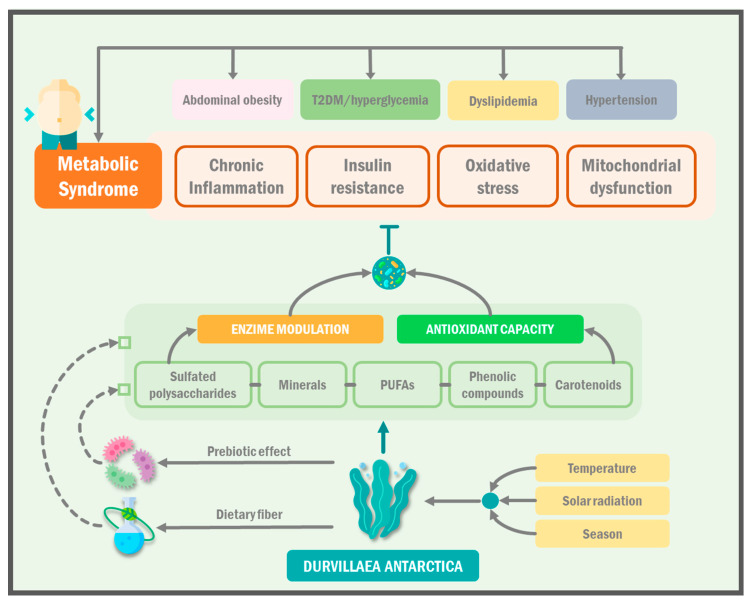
Control of metabolic syndrome of *Durvillaea antarctica*.

Imbalances in the blood lipid levels, specifically cholesterol, are characteristic of dyslipidemia, a condition that is commonly associated with metabolic syndrome. Cholesterol plays a vital role in maintaining cellular homeostasis by participating in the synthesis of hormones, bile acids, and membrane structures [66]. The regulation of whole-body cholesterol homeostasis involves tightly controlled processes, including de novo biosynthesis, dietary cholesterol absorption, and biliary clearance and excretion [67]. In this context, extracts from algae such as *D. antarctica* and *U. Lactuca* could play a beneficial role. It was observed that *U. Lactuca* samples are rich in polyunsaturated fatty acids (PUFAs), while *D. antarctica* samples show the highest content of saturated fatty acids (SFAs) [68]. Furthermore, significant concentrations of fatty acids were reported in macroalgae such as *Durvillaea antarctica*, with a higher content of monounsaturated fatty acids and PUFAs such as oleic acid (18:1n-9c) and linoleic acid (18:2n-6), particularly in the fronds of *D. antarctica* [69]. These algae extracts could be a promising alternative for managing dyslipidemia in metabolic syndrome due to their composition, which is rich in beneficial fatty acids for lipid profiles. However, further research is needed to evaluate their effectiveness and safety in clinical practice (Figure 4).

In relation to the pigment fucoxanthin, which has an antioxidant effect, it inhibits the differentiation of 3T3-L1 preadipocytes into adipocytes, decreasing weight gain and blood glucose concentration [70]. In addition, it increases lipolysis and thermogenesis by stimulating uncoupling protein 1 (UCP-1) and the β3-adrenergic receptor in white adipose tissue [71]. In rodents, it was observed that fucoxanthin increases the hepatic synthesis of docosahexaenoic acid, improving the lipid profile [72].

**Table 3:** A Comprehensive Summary: Exploring the Role of *Durvillaea antarctica* in cardiovascular health and metabolic syndrome.

Author (REF)	Phytochemical Compound Tested	Rationale	Results–Key Findings
Stiefvatter et al. [54]	*Phaeodactylum tricornutum* (PT)	Bioavailability and safety of consuming whole biomass of PT in humans. Intestinal health and microbiota were also assessed.	PT intake increased n-3 PUFA and EPA levels, decreased the n-6:n-3 ratio, and resulted in the uptake of fucoxanthinol (FX) and amarouciaxanthin A (A × A). No adverse effects were observed, supporting PT as a sustainable food source.
Chichibu et al. [55]	*Seaweed*	Seaweed intake was assessed through a 24 h dietary recall survey and categorized into four groups (0, 1–5.5, 5.5–15, and ≥15 g/day). The study examined the incidence of cardiovascular disease within the Circulatory Risk in Communities Study (CIRCS).	Seaweed intake was inversely associated with the risk of total stroke and cerebral infarction among men but not among women. The hazard ratios (95% confidence intervals) for the highest versus the lowest categories of seaweed intake were 0.63 (0.42–0.94; 0.01) for total stroke and 0.59 (0.36–0.97; 0.03) for cerebral infarction.
Kishida et al. [56]	*Seaweed*	Association between seaweed intake frequency and CVD mortality, including stroke subtypes and coronary heart disease, among Japanese participants in the Japan Collaborative Cohort Study for Evaluation of Cancer Risk.	Regular seaweed consumption was associated with lower hazard ratios for cardiovascular disease, stroke, and cerebral infarction in both men and women. The multivariable-adjusted hazard ratios were 0.72 (0.55–0.95; 0.001) for total cardiovascular disease, 0.70 (0.46–1.06; 0.01) for total stroke, and 0.49 (0.27–0.90; 0.22) for cerebral infarction.
Pacheco et al. [64]	*Durvillaea antarctica*, *Gelidium* sp., *Lessonia spicata*, *Nothogenia* sp., *Mazzaella laminarioides*, *Pyropia* sp.	Assess the anti-glycemic potential of seaweeds from southern Chile. HPLE was compared to acetone extraction for obtaining polyphenol-rich extracts for functional food development.	The acetone extract of *D. antarctica* had the highest TP content, while the HPLE ethanol/water extract exhibited the highest antioxidant activity. Cochayuyo extracts showed significant anti-enzymatic capacity against α-glucosidase and α-amylase. No extract affected cell viability.
Shih et al. [73]	*Durvillaea antarctica*	The potential of enzymatic hydrolysates from *D. antarctica* as natural antioxidants. Three hydrolysates, Dur-A, Dur-B, and Dur-C, were produced using viscozyme, cellulase, and α-amylase enzymes, respectively.	All of the following extracts demonstrated inhibitory effects on key enzymes related to metabolic syndrome: angiotensin I-converting enzyme (ACE), α-amylase, α-glucosidase, and pancreatic lipase. Dur-B showed superior antioxidant and anti-metabolic syndrome effects compared to the other extracts.

Abbreviations: n-3 polyunsaturated fatty acids (n-3 PUFAs); eicosapentaenoic acid (EPA); high-pressure liquid extraction (HPLE); total polyphenol (TP).

Illustration showcasing the potential health benefits of *Durvillaea antarctica* supplementation. Brown algae, which is rich in polyunsaturated fatty acids (PUFAs), minerals, polysaccharides, and polyphenols, can enhance the nutritional value of meals and improve lipid profile, heart disease, obesity, and comorbidities associated with metabolic syndrome. Mechanisms include the positive modulation of lipid metabolism enzymes, reduced thrombogenicity, and antioxidant properties that combat oxidative stress and mitochondrial dysfunction. Environmental factors affect algae’s composition and benefits. Incorporating marine algae, specifically *Durvillaea antarctica*, into the diet regulates hypertension, hyperglycemia, body weight, blood cholesterol, and cardiovascular diseases through the presence of PUFAs, dietary fiber, and antioxidants. T2DM: type 2 diabetes mellitus; PUFAs: polyunsaturated fatty acids.

Another preclinical study showed that three enzymatic hydrolysates from DA biomass, rich in sulfated polysaccharides, had a high antioxidant activity, as they were able to inhibit the activity of certain enzymes involved in human metabolism and whose function is exacerbated in the pathophysiology of MS, such as angiotensin I-converting enzyme (ACE), α-amylase, α-glucosidase, and pancreatic lipase [73].

Similarly, it was shown that the supplementation of brown algae that is rich in polyunsaturated fatty acids (PUFAs), minerals, polysaccharides, and polyphenols, very similar to those contained in DA, was associated with a greater nutritional value to the meals in which it was added, as well as improving the lipid profile, heart disease, and obesity, which are all variables or comorbidities associated with MS. The proposed mechanisms contributing to such pro-protective effects are the positive modulation of enzymes involved in lipid metabolism, a lower thrombogenic index, and antioxidant properties that are able to decrease the oxidative stress and mitochondrial dysfunction of crucial cells in carbohydrate metabolism, such as the pancreas and vascular endothelium [74,75,76].

It is important to mention that all of these previously mentioned benefits depend on the type of algae and the environmental conditions in which it is found (temperature, solar radiation, and season, among others) [77]. According to the information compiled in this review, the addition of marine algae to the diet, specifically *Durvillaea antarctica*, provides benefits in the regulation of hypertension and hyperglycemia and in the reduction in body weight and blood cholesterol, preventing possible cardiovascular diseases through the incorporation of polyunsaturated fatty acids, dietary fiber, and antioxidant compounds present in the algae [78].

It is essential to continue with research to clearly identify the effect of *Durvillaea antarctica* consumption on cardiovascular health since, in studies carried out to evaluate the effect of the consumption of brown marine algae, to which *Durvillaea antarctica* belongs, on metabolic syndrome, positive results were obtained in the reduction in the different factors that affect the development of MS (arterial hypertension, total cholesterol, and hyperglycemia) [74]. Among the negative effects related to the consumption of seaweed is the level of heavy metals that they could present, among which are cadmium, lead, silver, and arsenic [79].

## 2. Conclusions

*Durvillaea antarctica* consumption generates a positive impact on the immunological system by increasing the activation of CD19+B lymphocytes, promoting macrophages and anti-herpetic activities, and inducing the immunomodulatory effect via associated macrophage pathways. Additionally, an antioxidant activity was reported. In cardiovascular risk, it was described that the intake of *Durvillaea antarctica* has beneficial effects related to glycemic control and other metabolic parameters. This seaweed has high contents of n-3 fatty acid and n-6 essential fatty acid. Among its carotenoids, fucoxanthin stands out, which is involved in lipid metabolism and modulating the action of leptin and adiponectin, thus reducing lipogenesis and lipolysis and, therefore, decreasing cardiovascular risk.

The effect related to *D. antarctica* consumption in the regular diet on the microbiota is impressive. Specific nutrients such as β-glucans, polysaccharides (particularly fucoidan), laminarin, alginate, ulvan, and porphyran are unique to seaweeds. Several studies have shown their potential to act as prebiotics from the diet and positively modify and modulate the gut microbiota. At the same time, this probiotic effect improves the metabolic response, lowering weight gain and serum triglyceride concentration through their different bioactive substances.

Alginate, a polysaccharide extracted from *D. antarctica*, plays a crucial role in gut health. It promotes the growth of beneficial Bifidobacteria while inhibiting pathogenic bacteria, resulting in extended colonic fermentation. This process leads to a higher production of SCFAs. The sulphated polysaccharides present in seaweeds have anti-obesity, anti-inflammatory, anticancer, lipid-lowering, and hypoglycemic activities, due to their activity on the intestinal microbiota, promoting the relationship between Bacteroidetes and Firmicutes. The number of different groups of bacteria is crucial to maintain a healthy microbiota, and the function of the intestinal mucosal barrier is important in maintaining the balance of epithelial cells.

Bioactive peptides derived from marine algae, such as phycobiliproteins, glycoproteins, phycolectins, and mycosporine-like amino acids, exert beneficial effects. Through the stimulation of the epidermal growth factor, they promote the growth, proliferation, and differentiation of intestinal epithelial cells, thereby improving the health of the host. It is important to incorporate *Durvillaea antarctica* in the usual diet due to its functional bioactive components in the nutritional management of metabolic diseases, especially those associated with metabolic syndrome, as well as to improve the modulation of the immune response, and especially for its recognized benefits associated with potentiating intestinal health, promoting a healthy intestinal function in adults, and improving intestinal response in particular clinical conditions.

The present study provides compelling evidence of the potential benefits of *Durvillaea antarctica* in improving gut health, managing metabolic syndrome, and positively modulating the immune system. Our findings demonstrate that consuming *Durvillaea antarctica* can enhance the composition of the gastrointestinal microbiota, leading to better metabolic health outcomes. Moreover, the bioactive compounds present in *Durvillaea antarctica* possess immunomodulatory properties that may help prevent and treat various immune-related diseases. However, the primary limitation of our study is the lack of well-designed randomized clinical trials to fully evaluate the effects of *Durvillaea antarctica* on the gut microbiota. To gain a more comprehensive understanding of the potential health benefits of *Durvillaea antarctica*, future research should focus on conducting well-designed studies in this regard.

In order to fully explore the potential of *Durvillaea antarctica* extracts, further research should focus on investigating its comprehensive physicochemical properties. This includes identifying and characterizing the various phytochemicals present in the algae, which would allow for a thorough evaluation of its biological properties. It is essential to conduct not only preclinical studies, but also human studies, to assess the efficacy and safety of these extracts. Disseminating current knowledge and findings about *Durvillaea antarctica* among the scientific community is crucial to inspire and promote research in this field. By doing so, we can potentially uncover a valuable therapeutic tool against intestinal, immune, and, notably, cardiometabolic diseases. Future investigations should aim to provide a deeper understanding of the therapeutic potential of *Durvillaea antarctica* and its applications in various health conditions.

## Data Availability

No additional data were created during the analysis.
